# *Clonorchis sinensis* excretory/secretory proteins ameliorate inflammation in rheumatoid arthritis and ankylosing spondylitis

**DOI:** 10.1186/s13071-025-06677-3

**Published:** 2025-03-04

**Authors:** Moon-Ju Kim, Hee Min Yoo, Yu Jeong Lee, Hyun Hee Jang, Seung Cheol Shim, Eun Jeong Won, Tae-Jong Kim

**Affiliations:** 1 Department of Rheumatology, Chonnam National University Medical School and Hospital, Gwangju, Republic of Korea; 2 Biometrology Group, Korea Research Institute of Standards and Science (KRISS), Daejeon, 34113 Republic of Korea; 3 Department of Precision Measurement, University of Science and Technology (UST), Daejeon, 34113 Republic of Korea; 4 Department of Biomedical Sciences, Graduate School of Chonnam National University, Gwangju, 61469 Republic of Korea; 5 Division of Rheumatology, Daejeon Rheumatoid & Degenerative Arthritis Center, Chungnam National University Hospital, Daejeon, Republic of Korea; 6 Department of Laboratory Medicine, Asan Medical Center, University of Ulsan College of Medicine, Seoul, Republic of Korea

**Keywords:** *Clonorchis sinensis* excretory/secretory protein, Anti-inflammatory effects, RA, AS

## Abstract

**Background:**

We aimed to investigate whether substances secreted by *Clonorchis sinensis* excretory/secretory protein (CS-ESP) have an effect on the inflammation of rheumatoid arthritis (RA) and ankylosing spondylitis (AS) and to identify specific peptides through related proteomic analysis to determine which proteins exhibit anti-inflammatory effects more specifically.

**Methods:**

Peripheral blood mononuclear cells (PBMCs) were obtained from healthy controls (HCs), RA and AS patients. Cytotoxicity of CS-ESP at different doses was assessed by MTS and flow cytometry before performing experiments. Inflammatory cytokine producing cells were analyzed using flow cytometry. To determine the effect of CS-ESP in an arthritis mouse model, 8-week-old SKG mice were injected intraperitoneally with curdlan and treated with CS-ESP; body weight and paw swelling were checked twice a week. Inflammation was evaluated using immunohistochemistry. We conducted proteomic analysis on CS-ESP and identified specific Cs-GT and Cs-Severin proteins. In vitro effect of coculture with Cs-GT and Cs-Severin was determined by inflammatory cytokine measurements.

**Result:**

Treatment with CS-ESP resulted in no reduced cell viability of PBMCs. In experiments culturing PBMCs, the frequencies of IL-17A and GM-CSF producing cells were significantly reduced after CS-ESP treatment. In the SKG mouse model, CS-ESP treatment significantly suppressed clinical score, arthritis and enthesitis. Treatment with Cs-GT and Cs-Severin resulted in no reduced cell viability of HC PBMCs. After Cs-GT and Cs-Severin treatment of HC PBMC, the frequencies of IL-17A and GM-CSF producing cells were significantly reduced.

**Conclusions:**

We provide evidence showing that CS-ESP, Cs-GT and Cs-Severin can ameliorate clinical signs and cytokine derangements in AS.

**Graphical Abstract:**

**Figure:**
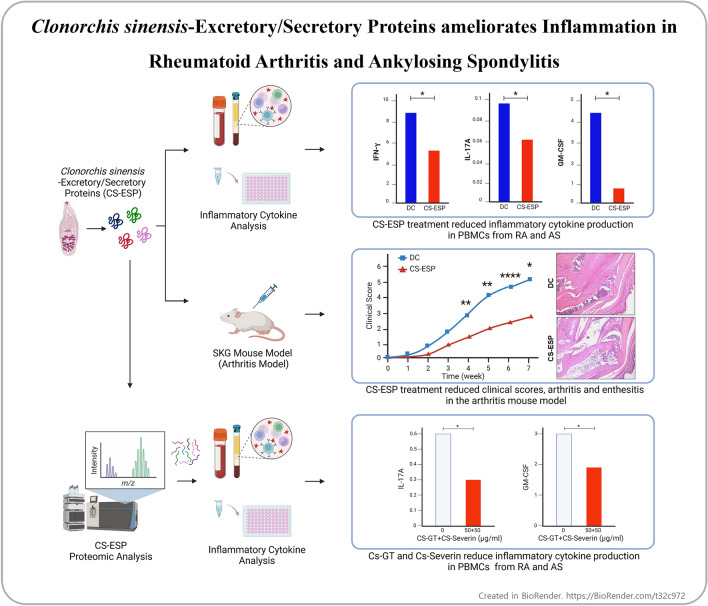

## Background

Inflammatory rheumatoid diseases (IRDs) include various conditions including rheumatoid arthritis (RA), ankylosing spondylitis (AS), systemic lupus erythematosus (SLE) and Bechet’s disease (BD) [1]. Notably, the chronic, progressive nature of RA and AS contributes to an inflammatory process, resulting in joint damage, and a decline in the patient’s physical health RA is characterized by synovial proliferation, angiogenesis, inflammatory cell infiltration, pannus formation, cartilage destruction and bone erosion, which can lead to joint destruction and disability as the disease progresses [2]. AS mainly affects the spine and ceiling joints and can affect the eyes, heart, lungs and other joints. Tumor necrosis factor (TNF) plays a major role in the pathogenesis of AS [3], and administering TNF inhibitors that inhibit it is effective in the treatment of RA and AS [4, 5]. However, despite these developments, TNF inhibitors are still not the optimal treatment as they can cause various side effects. Thus, a novel therapeutic strategy for RA and AS is needed. Recently, many attempts have been made to use parasite administration, such as ingestion of eggs of the nematode *Trichuris suis*, as a new way to treat autoimmune diseases, and the results of some clinical trials have shown potential therapeutic effectiveness [6]. We previously investigated the therapeutic effect of *Clonorchis sinensis*-derived protein (CSp) on AS using a mouse model [7]. Although the beneficial effect of CSp was first clarified as inhibiting new bone formation, which was one the of substantial clinical challenges in that study, using crude extracts was the main hurdle to further standardization of the procedure. This led us to investigate whether substances secreted by *C. sinensis* excretory/secretory protein (CS-ESP), rather than direct contact with CSp, have an effect on inflammation in RA and AS and to identify specific proteins through related proteomic analysis to determine which proteins exhibit anti-inflammatory effects more specifically.

## Methods

### Human samples

All patients fulfilled the RA criteria established by the American College of Rheumatology/European League Against Rheumatism. All participants satisfied the modified New York criteria for AS. Peripheral blood mononuclear cells (PBMCs) were collected from individuals classified as healthy controls (HC) as well as those diagnosed with RA and active AS. We investigated according to the principles set out in the Declaration of Helsinki and approved by the ethics committee of Chonnam National University Hospital (CNUH). Written informed consent was obtained from all participants (IRB no. CNUH-2011-199).

### *Clonorchis sinensis* excretory/secretory protein (CS-ESP) preparation

After infection by feeding *C. sinensis* eggs to rabbits, adult worms were obtained as previously described [8]. All experiments were approved by the Institutional Animal Care and Use Committee (CNU IACUC-H-2018-35). They were conducted under the Laboratory Animals Welfare Act, Guide for the Care and Use of Laboratory Animals. The preparation of CS-ESP was performed as previously described [9]. Briefly, the obtained adult worms were pre-cultured for 1 h in PBS containing 1 × protease inhibitor cocktail, and then the adult worms were transferred to new PBS and cultured for 3–4 h with the addition of 1 × penicillin (200 U/ml) and streptomycin (200 U/ml) and 100 μM of cholic acid at 37 °C in 5% CO_2_. The culture was centrifuged, and the supernatants containing the CS-ESPs were filtered through a 0.2-μm filter, freeze-dried and dissolved in 1X PBS, and the concentration was measured using the Pierce™ BCA Protein Assay Kit (Thermo Scientific Co., Rockford, IL, USA) and aliquoted for storage at − 80 °C.

### Protein preparation and digestion

Proteins were quantified using the BCA assay, and digestion was performed following the Filter-Aided Sample Preparation (FASP) protocol. Proteins were reduced with 5 mM TCEP (Sigma-Aldrich, St. Louis, MO, USA) at 37 °C for 30 min and alkylated with 50 mM IAA (Sigma-Aldrich, St. Louis, MO, USA) in the dark at 25 °C for 1 h. Buffer exchange was carried out using 8 M urea (Sigma-Aldrich, St. Louis, MO, USA) and 50 mM ammonium bicarbonate (ABC; Sigma-Aldrich, St. Louis, MO, USA). Proteins were digested with trypsin at a 1:50 enzyme-to-protein ratio at 37 °C for 18 h, and digestion was terminated by adding formic acid (Sigma-Aldrich, St. Louis, MO, USA) adjusted to pH 2.

### Peptide desalting

Peptides were desalted using C18 Micro Spin-Columns pre-equilibrated with 80% acetonitrile (ACN; Sigma-Aldrich, St. Louis, MO, USA) and 0.1% trifluoroacetic acid (TFA; Sigma-Aldrich, St. Louis, MO, USA). Peptides were loaded, washed with 0.1% TFA and eluted sequentially with 80% ACN and 100% ACN. Desalted peptides were dried using a SpeedVac and stored at − 20 °C until analysis.

### LC-MS/MS analysis

Peptides were analyzed using an LC-MS/MS system equipped with a C18 trapping column and an analytical PepMap™ RSLC C18 column (Thermo Fisher Scientific, Waltham, MA, USA). Mobile phases consisted of 0.1% formic acid in water as Phase A and 80% ACN with 0.1% formic acid as Phase B. A gradient of 4–40% solvent B was applied over 120 min at a flow rate of 300 nl/min. Mass spectra were acquired in the range of 400–2000 m/z.

### Data analysis

LC-MS/MS data were analyzed using Proteome Discoverer software (Thermo Fisher Scientific, Waltham, MA, USA) with the Uniprot database for *Clonorchis sinensis*. Modifications including acetylation, oxidation, carbamylation and carbamidomethylation were applied for protein identification. The mass spectrometry proteomics data were deposited at the ProteomeXchange Consortium via the PRIDE partner repository with the dataset identifier PXD059467.

### Selection and purification of Cs-GT and Cs-Severin protein

The coding sequences of Cs-GT (accession no. A0A3R7DB39) and Cs-Severin (accession no. A0A419QFQ4) were codon optimized and abundantly expressed in strain BL21 (DE3) transformed with the recombinant plasmid of pET-28b (+)-Cs-GT or Cs-Severin by 1.0 mM of isopropyl β-d-thiogalactopyranoside (IPTG) induction. The optimal conditions for soluble protein expression of Cs-GT and Cs-Severin were determined to be 37 °C, 1.0 mM IPTG and 4 h of induction, as this resulted in the highest soluble protein fraction (70%). The recombinant protein was purified using nickel-nitrilotriacetic acid (Ni–NTA) affinity chromatography and performed with elution by 300 mM imidazole. The protein concentration was determined by BCA Protein Assay Kit. All the protein was sterilized by the 0.22-μm filters before being used in animal experiments.

### Bioinformatics analysis

The identified proteins were subjected to Gene Ontology (GO) enrichment analysis to categorize them based on their biological processes (BP), molecular functions (MF), and cellular components (CC). This analysis was performed using the DAVID bioinformatics resources, focusing on significantly enriched GO terms. Heatmaps were generated to visualize the scaled abundance of proteins and their associated GO terms across the different samples. The color scale represents the relative abundance of each protein, with values ranging from –1.5 (underrepresented) to 1.5 (overrepresented). The heatmap was divided into sections corresponding to protein abundance in CS-ESP, PEL and SUP as well as their association with specific GO terms in BP, MF and CC categories.

### Cell viability assay

The cells were seeded at a density of 5 × 10^4^ cells/well in 100 μl culture medium per well and treated with various concentrations of CS-ESP (24, 48 and 72 h), Cs-GT and Cs-Severin (24 h), and cell viability was assessed using Cell Titer 96 AQueous One Solution Reagent (G3580, Promega, USA). Following the manufacturer’s instructions, 20 μl of MTS solution was added to 100 μl of the cell culture medium and incubated at 37 °C for 2 to 4 h. Absorbance was measured at 490 nm using a Reader 96-well microplate reader (Molecular Devices, USA). Live cells were surface stained with anti-Fixable Viability Dye-eFluor780 (65–0865-14, Invitrogen, USA), and cell viability was analyzed by flow cytometry.

### Co-culture of human inflammatory cells with CS-ESP and proteins

PBMCs were isolated and suspended in a complete medium (RPMI 1640, 2 mM l-glutamine, 100 units/ml penicillin, and 100 μg/ml streptomycin) supplemented with 10% fetal bovine serum (FBS; Gibco BRL, Grand Island, NY, USA). Cells were seeded into 96-well plates at a density of 1 × 10^6^ cells/well. After a 3-h pretreatment with CS-ESP, Cs-GT and Cs-Severin, the cells were stimulated with phorbol 12-myristate 13-acetate (PMA; 100 ng/ml, P1585, Sigma, USA), ionomycin (1 μM, I9657, Sigma, USA) and brefeldin A (555029, BD, USA) cultured in a CO_2_ incubator at 37 °C for 4 h. Cells were stained with Pacific Blue-conjugated anti-CD4 (300521, BioLegend, USA) and anti-Fixable Viability Dye-eFuor780 (65-0865-14, Invitrogen, USA). After washing, cells were fixed and permeabilized using perm/washing buffer. They were stained with FITC Mouse anti-human IFN-r (552887, BD, USA), APC-conjugated anti-IL-17A (512334, BioLegend, USA) and PerCP/Cyanine5.5 anti-human GM-CSF (502312, BioLegend, USA) and analyzed using FlowJo Software (BD, USA).

### Experimental mouse model and clinical scoring

The experiment was conducted with institutional Animal Care and Use Committee (animal experiment IRB no. IACUC-H-2019-36) approval. SKG mice on a BALB/c background were purchased from Clea Japan, Inc. (Tokyo, Japan) and bred in a specific pathogen-free (SPF) condition. In this study, 8-week-old female mice were used, and the experiment had three groups: a negative control (NC) group (*n* = 6 mice), a disease control (DC) group (*n* = 8 mice) and a CS-ESP treatment group (*n* = 9 mice). To assess baseline responses, negative control mice were not injected with curdlan. For both DC and CS-ESP treatment groups, mice were administered curdlan (Wako, Osaka, Japan) at 3 mg/kg intraperitoneally (i.p.). In the CS-ESP treatment group, CS-ESP (10 μg/kg) was administered i.p. twice a week from 3 weeks after arthritis induction until sacrifice. Clinical scoring of the affected joints was summed following the previous report by Ruutu et al. [10]. Joint scoring was as follows: 0 = asymptomatic; 0.1 = swelling per toe; 0.5 = swelling of the ankle; 1 = severe swelling of the ankle. The highest possible score per mouse was 6 points. Clinical scoring was performed twice a week.

### Histological analysis

Hematoxylin and eosin, safranin O and toluidine blue method was used to confirm histological differences. After the experiment, mice were killed, and ankle samples were collected and fixed in 10% formalin for 1 week. They were decalcified with 10% formic acid and embedded in paraffin blocks. Paraffin blocks were cut at 3.5 μm thickness and stained according to the standard protocols. Histological scores of joints were summed according to previous reports, and pathological scoring was performed by two blind readers. Histological scoring for arthritis was also conducted by two blinded readers following the same reference by Ruutu et al. [10]. Joint histological features were evaluated using a scale of 1–4: 1 = few infiltrating immune cells; 2 = 1–2 small patches of inflammation; 3 = inflammation throughout the ankle joint; 4 = inflammation in soft tissue/entheses/fasciitis. The histological scoring system for enthesitis was adapted from Benham et al. [11]. The criteria were as follows: 1 = mild inflammation at tendon insertion site; 2 = mild-to-moderate inflammatory infiltrate at the insertion site and along the tendon; 3 = severe inflammation with bone involvement; 4 = severe inflammation with obliteration of the tendon-bone interface.

### Statistical analysis

Data were analyzed with GraphPad Prism 10 software (GraphPad Software, San Diego, CA, USA). Differences between means were evaluated for statistical significance using various tests, including Kruskal-Wallis test with Dunn’s multiple comparisons, *t*-test, Wilcoxon matched-pairs signed-rank test, two-way analysis of variance (ANOVA) and Mann-Whitney test. *P* < 0.05 was considered a statistically significant difference: *P < 0.05; **P < 0.01, ***P < 0.001; ****P < 0.0001.

## Result

### CS-ESP does not affect cell viability

**Fig. 1:**
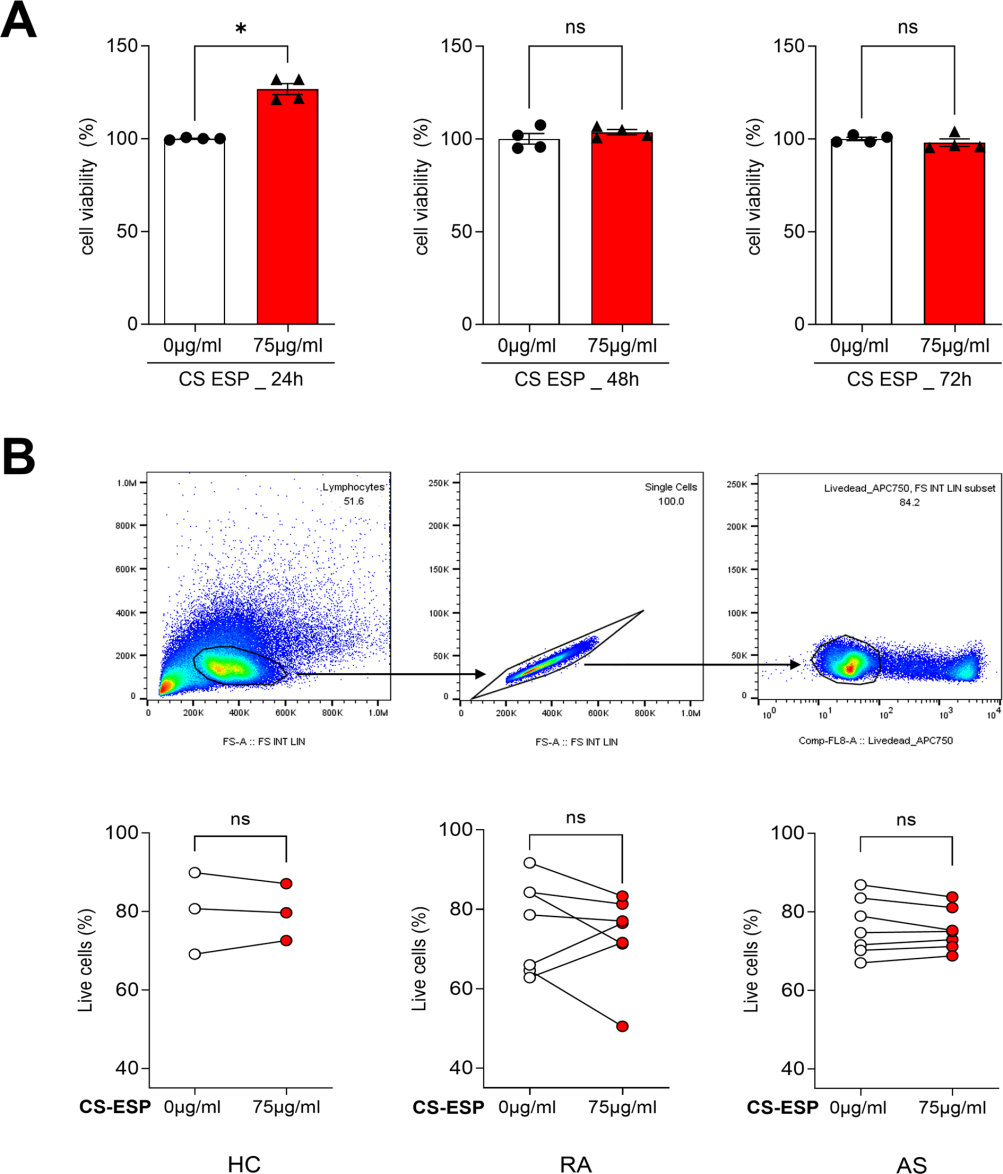
Assessment of cell viability upon CS-ESP treatment. **A** The viability of PBMC was evaluated using the MTS assay for each CS-ESP concentration. Kruskal-Wallis test with Dunn’s multiple comparisons was performed to determine statistical significance. Values are the mean ± SD. **B** HC, RA and AS PBMCs cell viability was measured by flow cytometry. A representative gating strategy for flow cytometry was employed to evaluate the survival rate (upper panel). Viability dyes of PBMCs were stained and measured (lower panel). Mann-Whitney test was performed to determine statistical significance. Values are the mean ± SD. Symbols represent the individual sample. CS-ESP: *Clonorchis sinensis* excretory/secretory protein; ns: not significant

PBMCs were treated with 75 μg/ml of CS-ESP, and cell viability was analysis by MTS. There was no effect on cell viability until 72 h (Fig. 1A). This suggests that CS-ESP is safe regarding cell viability. To verify these results, cell viability was also assessed using flow cytometry. Flow cytometry was performed on PBMCs from HC as well as RA and AS patients. The results corroborate the observation of the MTS assay, indicating that CS-ESP did not affect cell viability in PBMCs in HC, RA and AS patients (Fig. 1B).

### CS-ESP exhibits significant inhibition of inflammatory cytokines in PBMCs

**Fig. 2:**
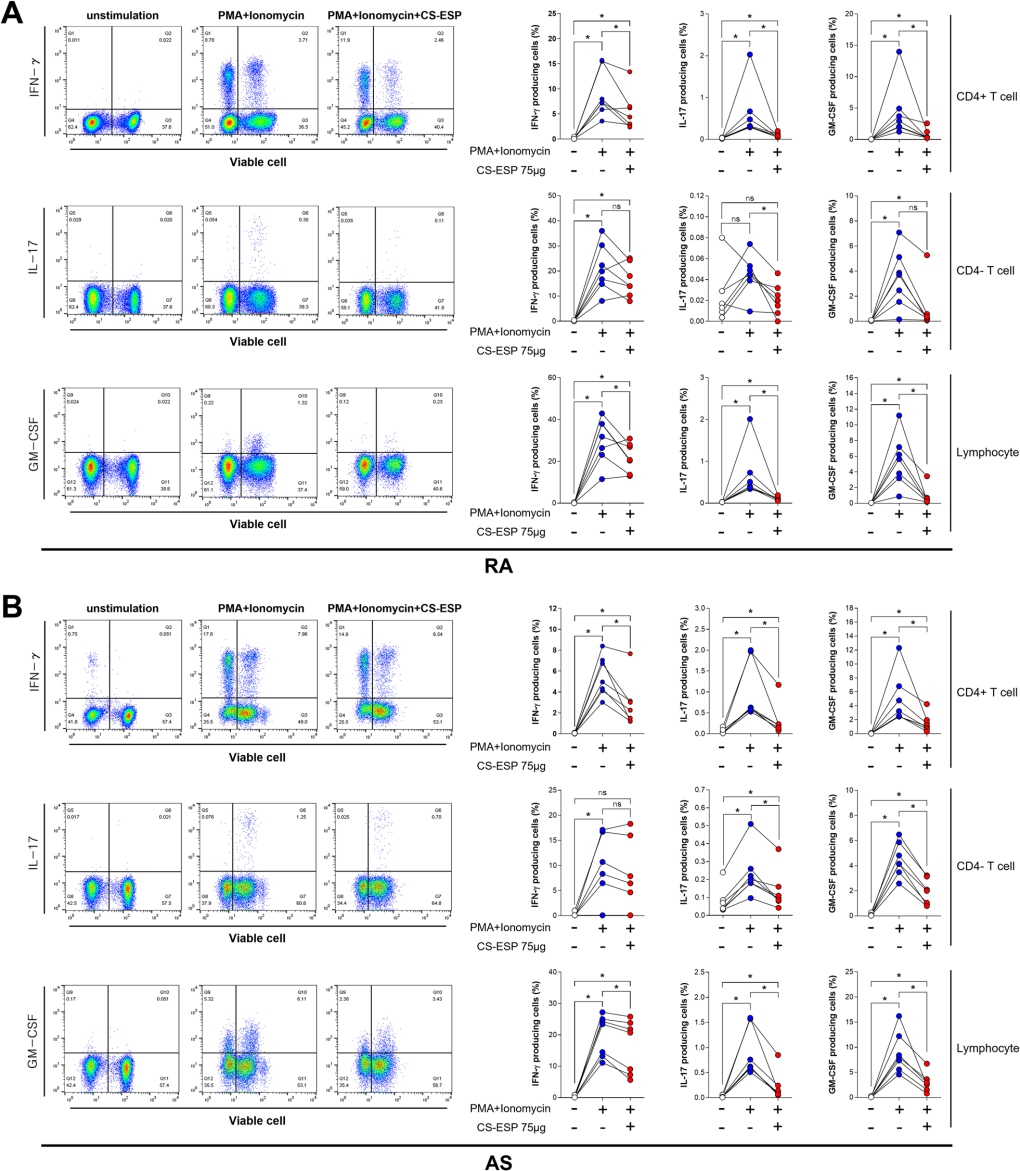
Treatment of CS-ESP inhibits inflammatory cytokine production in PBMCs from both **A** RA and **B** AS patients. Cells were pretreated for 3 h in the presence or absence of CS-ESP 75 μg/ml treatment, and then cells were stimulated with PMA 100 ng/ml and ionomycin 1 µM for 4 h. The representative flow cytometric features are shown (left panels of **A** and **B**, respectively). We present an analysis of the percentage of INF-γ-, IL-17A- and GM-CSF-positive cells obtained from PBMCs from patients with RA and AS (right panels of **A** and **B**, respectively). Mann-Whitney test was performed to determine statistical significance. Symbols represent the individual sample. *P < 0.05. ns: not significant

To investigate the anti-inflammatory effect of CS-ESP, PBMCs obtained from active AS and RA patients were stimulated and cultured ex vivo for 7 h in the presence or absence of CS-ESP. The frequencies of IFN-γ, IL-17A and GM-CSF producing cells were analyzed using flow cytometry. In RA PBMCs, frequencies of interferon-gamma (IFN-γ) (9.0 ± 4.73 vs. 5.5 ± 3.83, *P* = 0.0313), interleukin-17A (IL-17A) (0.6 ± 0.63 vs. 0.1 ± 0.05, *P* = 0.0156) and granulocyte-macrophage colony-stimulating factor (GM-CSF) (4.4 ± 4.4 vs. 0.7 ± 0.87, *P* = 0.0156) producing cells in CD4-positive T cells were significantly reduced after treatment with CS-ESP (Fig. 2A upper panels). The frequencies of IL-17A (0.04 ± 0.02 vs. 0.02 ± 0.01, *P* = 0.0156; 0.7 ± 0.60 vs. 0.1 ± 0.05, *P* = 0.0156, respectively) and GM-CSF (3.4 ± 2.3 vs. 1.0 ± 1.89, *P* = 0.0156; 5.4 ± 3.30 vs. 0.8 ± 1.19, *P* = 0.0156, respectively) producing cells in CD4-negative T cells and lymphocytes decreased significantly after CS-ESP treatment, and IFN- γ tended to decrease (Fig. 2A middle and lower panels). In AS PBMC, the frequency of IFN-γ (5.5 ± 1.91 vs. 2.9 ± 2.27, *P* = 0.0156; 19.8 ± 6.61 vs. 16.3 ± 8.60, *P* = 0.0156, respectively), IL-17A (1.0 ± 0.69 vs. 0.3 ± 0.39, *P* = 0.0156; 0.9 ± 0.48 vs. 0.2 ± 0.28, *P* = 0.0156, respectively) and GM-CSF (5.0 ± 3.61 vs. 1.5 ± 1.31, *P* = 0.0156; 8.9 ± 4.04 vs. 2.9 ± 1.91, *P* = 0.0156, respectively) producing cells was significantly reduced in CD4-positive T cells and lymphocytes after CS-ESP treatment (Fig. 2B upper and lower panels). In CD4-negative T cells, the frequency of IL-17A (0.2 ± 0.13 vs. 0.1 ± 0.11, *P* = 0.0156) and GM-CSF (4.5 ± 1.35 vs. 1.9 ± 1.03, *P* = 0.0156) producing cells was significantly reduced, and IFN-γ tended to decrease, although there was no statistical difference (Fig. 2B middle panels).

### CS-ESP alleviate arthritis in the SKG mice

**Fig. 3:**
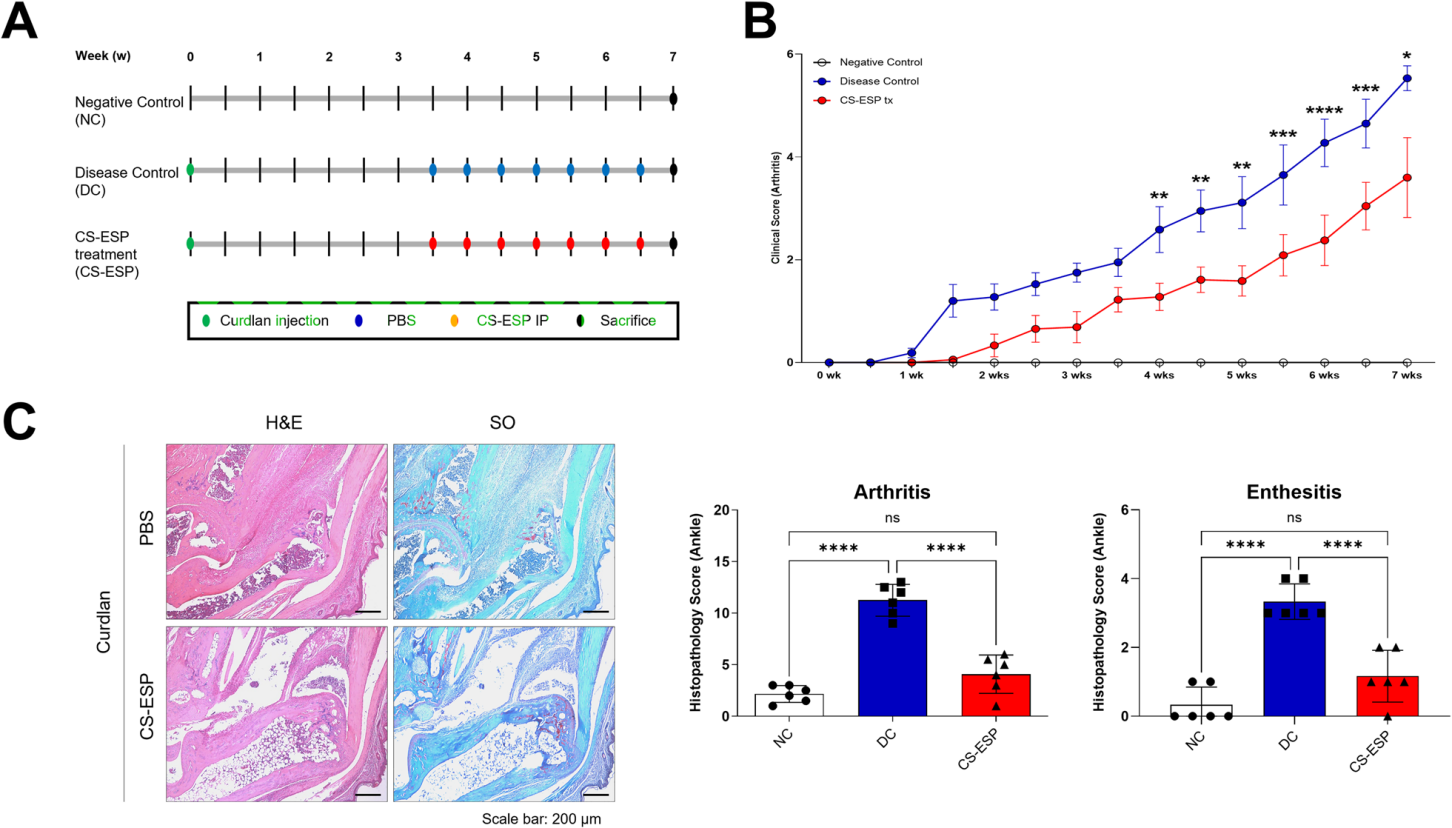
CS-ESP treatment reduces arthritis and enthesitis in SKG mice. **A** SKG mice were injected i.p. with curdlan, and 3.5 weeks later, treatment with CS-ESP or vehicle was performed i.p. **B** Arthritis score was determined based on clinical arthritis severity in each group. Two-way analysis of variance (ANOVA) was performed to determine statistical significance for the clinical score. Values are the mean ± SEM. **C** A representative tissue stain with H&E and safranin O of the ankle joint at the end of the experiment is displayed (left panels). Symbols represent the individual sample. Analysis of histological scores for arthritis and enthesitis were shown in bar graphs (right panels). Mann-Whitney test was performed to determine statistical significance. *P < 0.05, **P < 0.01, ***P < 0.001, ****P < 0.0001

To investigate the effect of CS-ESP on arthritis progression in an in vivo model, SKG mice were administered CS-ESP 3.5 weeks after curdlan injection (Fig. 3A). CS-ESP treatment effectively delayed the development of arthritis and significantly reduced its severity. These results were consistent throughout the experiment (Fig. 3B). At the end of the experiment, CS-ESP injection significantly suppressed the arthritis symptoms (5.5 ± 0.42 vs. 3.6 ± 1.56, *P* = 0.0116). This reduction in clinical scores underscored the potential of CS-ESP as an anti-inflammatory agent. Representative heel tissue stains at the end of the experiment are shown (Fig. 3C left panels). Histological evaluation showed reduced arthritis (NC vs. DC: 2.2 ± 0.82 vs. 11.3 ± 1.54, *P* < 0.0001; DC vs. CS-ESP: 11.3 ± 1.54 vs. 4.1 ± 1.86, *P* < 0.0001) and enthesitis (NC vs. DC: 0.3 ± 0.52 vs. 3.3 ± 0.52, *P* < 0.0001; DC vs. CS-ESP: 3.3 ± 0.52 vs. 1.2 ± 0.75, *P* < 0.0001) in mice treated with CS-ESP compared to DC mice (Fig. 3C right panels). These histopathological findings corroborate the clinical scoring data and indicate a specific anti-inflammatory effect of CS-ESP at the tissue.

### Proteins identified among the CS-ESPs

**Fig. 4:**
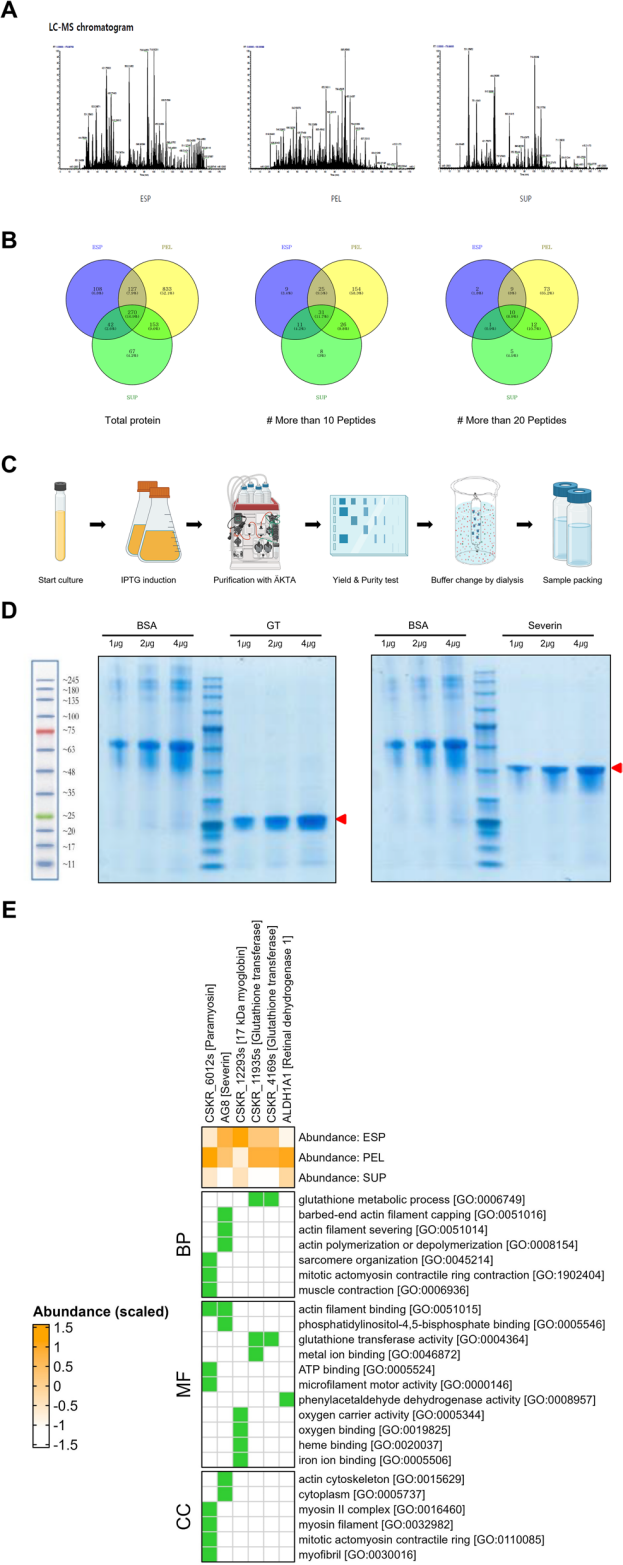
CS product proteomics analysis and peptide production. **A** Proteomic analysis of CS protein, pellet and CS-ESP using LC-MS chromatogram. **B** After the protein analysis of CS product, the selection of candidate groups was shown in a venn diagram. **C** Process overview of protein expression and purification. **D** Recombinant protein images of selected peptides Cs-GT (left panels) and Cs-Severin (right panels). **E** Scaled protein abundance and functional enrichment across different fractions in *Clonorchis sinensis*. The heatmap displays the scaled abundance of proteins in three fractions: CS-ESP, PEL and SUP, with a color scale ranging from –1.5 (underrepresented) to 1.5 (overrepresented). Proteins such as paramyosin, severin and multiple glutathione transferases show distinct expression patterns across these fractions. Functional enrichment analysis is illustrated for biological processes (BP), molecular functions (MF) and cellular components (CC), revealing the roles and regulatory mechanisms of proteins in different *C. sinensis* fractions

CS-ESPs were obtained by culturing worms for a period of 6 h in PBS. The proteomic characterization of this mixture yielded 1383 unique proteins to be identified (Fig. 4). Using peptide spectrum matches (PSMs) to estimate the relative abundance of these proteins, the top five most abundant and characterized proteins were identified as glutathione transferase, cathepsin L, calpain, severin and thioredoxin. Additionally, proteases such as retinal dehydrogenase 1, glyceraldehyde-3-phosphate dehydrogenase, cytidine deaminase and glutamate dehydrogenase were among the top 20 most abundant proteins. A Venn diagram comparing the proteins identified in the CS-ESP, pellet (PEL) and supernatant (SUP) fractions showed that 547 proteins were found in the CS-ESP fraction, 1383 in the PEL fraction and 532 in the SUP fraction. After selecting proteins identified by > 20 peptides, 10 proteins were common to all three fractions, 19 were found in both the CS-ESP and PEL fractions, 11 in both the CS-ESP and SUP fractions and 22 in both the PEL and SUP fractions.

### Protein abundance across different fractions among *C. sinensis*

The heatmap in Fig. 4E displays the scaled abundance of proteins in three fractions: CS-ESP, PEL and SUP. The color scale indicates relative protein abundance, ranging from –1.5 (underrepresented) to 1.5 (overrepresented). In the CS-ESP fraction, paramyosin was moderately overrepresented, while Cs-Severin and Cs-GT were significantly overrepresented. In the PEL fraction, most proteins showed balanced to slight underrepresentation, with two glutathione transferases being slightly overrepresented. The SUP fraction exhibited a high overrepresentation of paramyosin, and glutathione transferases were moderately represented in this fraction.

Functional enrichment analysis of biological processes (BPs) revealed significant overrepresentation of proteins involved in glutathione metabolic processes, actin filament capping and severing, actin polymerization or depolymerization, sarcomere organization and muscle contraction. In terms of molecular functions (MFs), enriched functions included actin filament binding, glutathione transferase activity, metal ion binding, ATP binding and microfilament motor activity. Cellular component (CC) analysis showed significant involvement of the actin cytoskeleton, cytoplasm, myosin II complex, myosin filaments and myofibrils.

### CS-ESP proteins exhibit significant inhibition of inflammatory cytokines in PBMCs

**Fig. 5:**
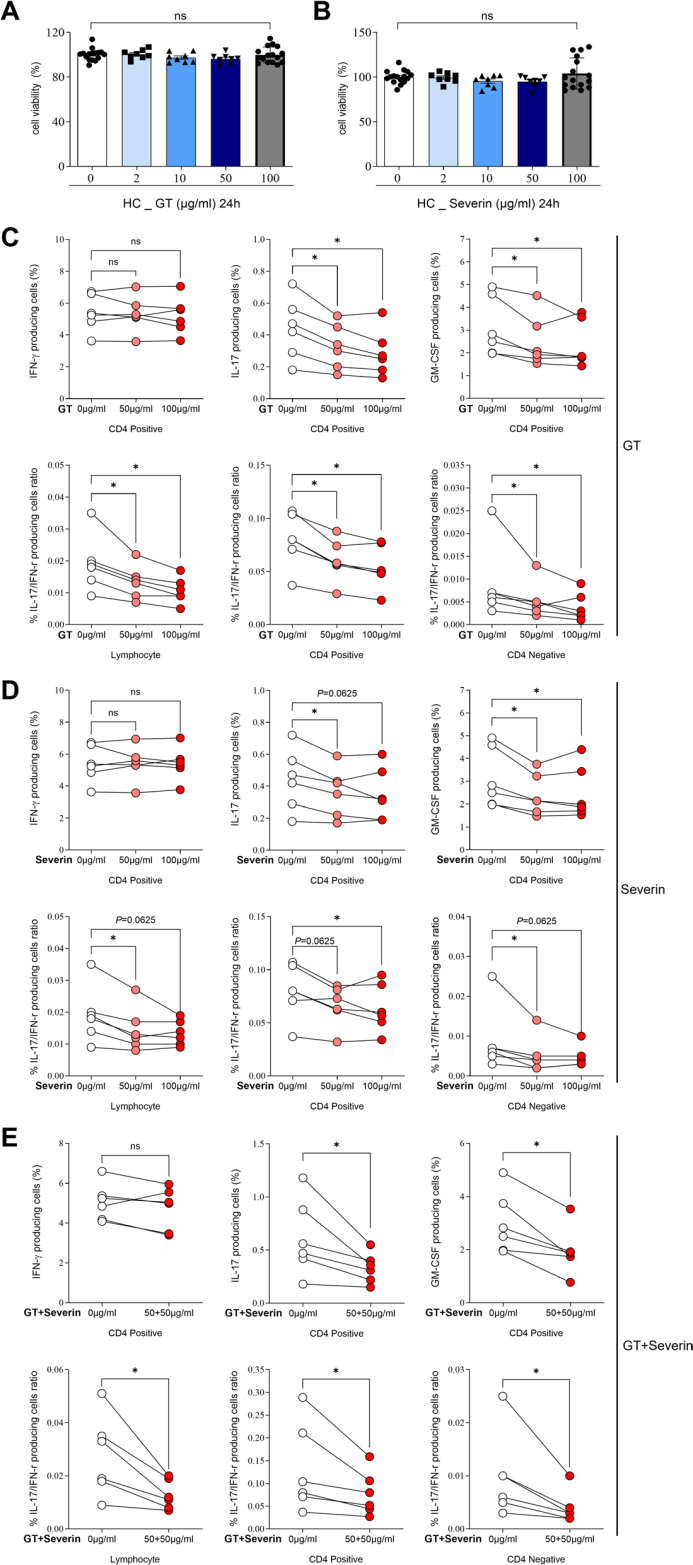
Treatment of Cs-GT and Cs-Severin inhibits inflammatory cytokine production in PBMCs from healthy controls. PBMC viability was evaluated using the MTS assay with Cs-GT (**A**) and Cs-Severin (**B**) concentrations of up to 100 μg/ml. Cells were pretreated for 3 h in the presence or absence of protein treatment, and then cells were stimulated with PMA 100 ng/ml and ionomycin 1 µM for 4 h. PBMCs were treated with 50 and 100 μg/ml of Cs-GT (**C**), 50 and 100 μg/ml of Cs-Severin (**D**) and 50 μg/ml of Cs-GT and Cs-Severin simultaneously (**E**) and then analyzed for the percentage of INF-γ, IL-17A and GM-CSF CD4-positive positive cells. Mann-Whitney test was performed to determine statistical significance. Symbols represent the individual sample. *P < 0.05; ns: not significant

**Fig. 6:**
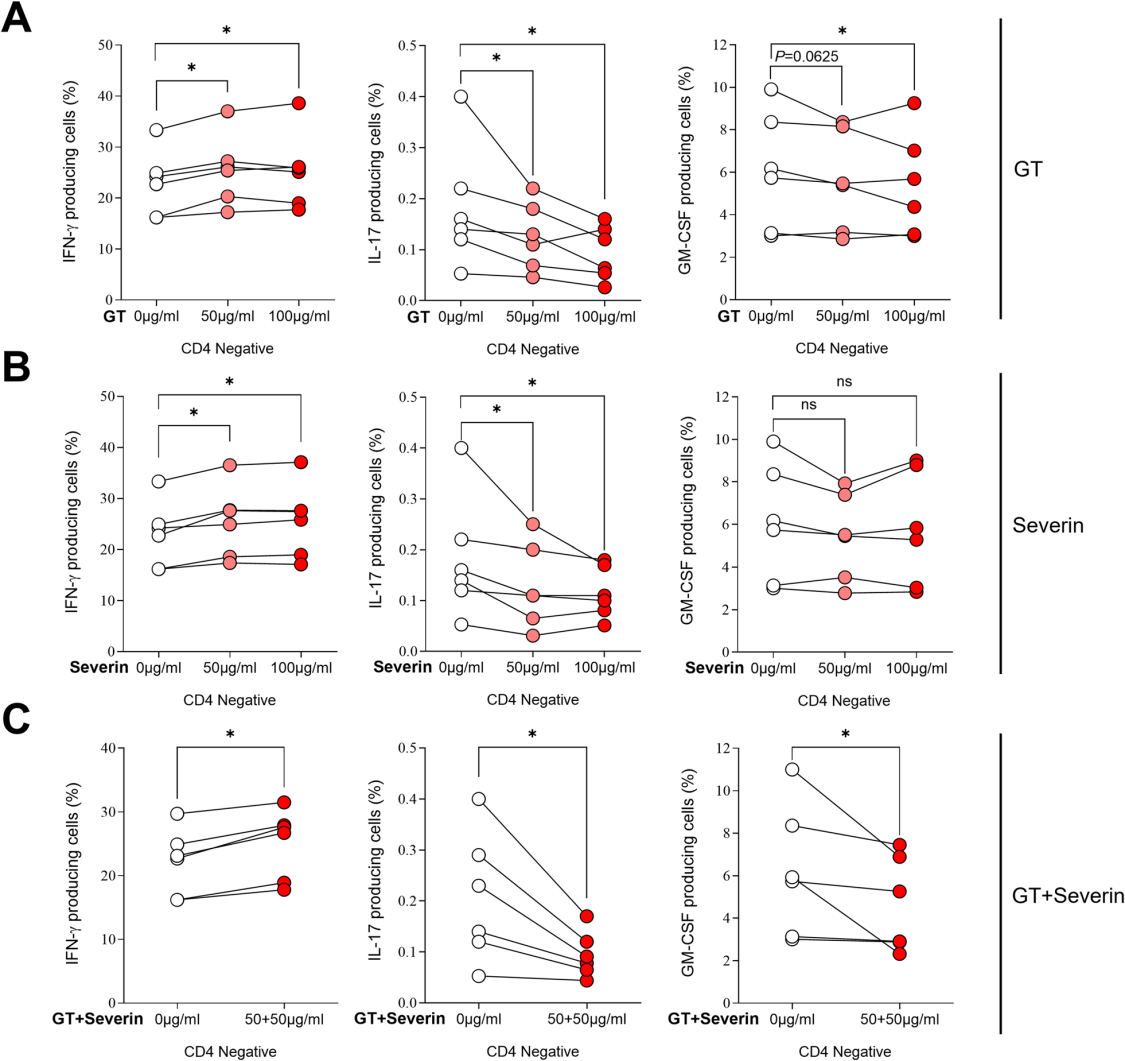
Treatment with Cs-GT and Cs-Severin inhibits inflammatory cytokine production in PBMCs from healthy controls. Cells were pretreated for 3 h with or without the proteins followed by stimulation with 100 ng/ml PMA and 1 µM ionomycin for 4 h. **A** PBMCs were treated with 50 and 100 μg/ml of Cs-GT, Cs-Severin, or a combination of Cs-GT and Cs-Severin and analyzed for the percentage of IFN-γ, IL-17A and GM-CSF producing CD4-negative cells. **B** The IL-17A/IFN-γ ratio was evaluated for Cs-GT, Cs-Severin and the combination treatment in total PBMCs, CD4-positive and CD4-negative cells. The Mann-Whitney test was used to assess statistical significance. Symbols represent individual samples. *P < 0.05; ns: not significant

To investigate the anti-inflammatory effects of CS-ESP proteins Cs-GT and Cs-Severin, HC PBMCs were stimulated and cultured in vitro for 7 h in the presence or absence of Cs-GT (50–100 μg/ml), Cs-Severin (50–100 μg/ml) and Cs-GT/Cs-Severin combination (50 μg/ml each). Before conducting these experiments, we measured cell viability for Cs-GT and Cs-Severin, but neither protein had any impact on cell viability (Fig. 5A, B). The frequencies of IFN-γ, IL-17A and GM-CSF producing CD4-positive cells were analyzed using flow cytometry. In HC PBMCs, frequencies of IL-17A and GM-CSF producing cells were significantly reduced after treatment with Cs-GT 50 and 100 μg/ml (Fig. 5C). Upon treatment with Cs-Severin, IL-17A decreased significantly at 50 μg/ml (0.4 ± 0.19 vs. 0.4 ± 0.15, *P* = 0.0313) and tended to decrease at 100 μg/ml. GM-CSF decreased by both 50 and 100 μg/ml (Fig. 5D). Combined treatment with 50 µg/ml each of Cs-GT and Cs-Severin resulted in a reduction of IL-17A (0.6 ± 0.36 vs. 0.3 ± 0.14, *P* = 0.0313) and GM-CSF (3.0 ± 1.15 vs. 1.9 ± 0.89, *P* = 0.0313) (Fig. 5E). We also performed an analysis on CD4-negative cells. In these cells, treatment with Cs-GT and Cs-Severin resulted in a reduction in IL-17A and GM-CSF levels, while IFN-γ showed a tendency to increase (Fig. 6). Further studies are required to explore the underlying mechanisms of this specific cellular response. However, when considering the IL-17A/IFN-γ ratio across total PBMCs, as well as CD4-positive and CD4-negative cells, an overall decrease was observed, indicating a general anti-inflammatory pattern (lower panel of Fig. 5C–E).

## Discussion

Helminth parasites have developed strategies to survive for long periods within immune-competent hosts by inducing strong anti-inflammatory or immune-modulating responses. An inverse relationship was observed between autoimmune diseases and helminth infections, suggesting that helminth infections may play a protective role in various autoimmune diseases [12]. Several studies demonstrated the potential of helminth derivatives as preventive and therapeutic agents for immune diseases in mouse models of RA and SLE [13, 14]. Through previous research, we discovered that a parasite, CS-ESP, improves clinical symptoms and cytokine abnormalities of AS and reduces new bone formation in an in vivo mouse model of AS [7]. However, no studies have confirmed the anti-inflammatory effect of CS in RA, and research on further characterization of CS-ESP is still lacking. CS-ESPs are antigens that include a complex mixture of secreted proteins and some extracellular vesicles (EVs) secreted through the parasite’s tegument and products from oral openings or the parasite's gut. It is well known that CS-ESPs can cause histopathological changes such as bile duct dilatation, inflammation and fibrosis, and adenomatous proliferation of the biliary epithelium [15]. The components of CS-ESPs also have value in rendering a diagnosis [16, 17]; however, the therapeutic molecule remains obscure. Identification of the composition of the ESPs could provide attractive materials for identifying causative agent candidates and new drug targets. In this study, we demonstrated the protective effect of CS-ESP itself on AS and then suggested two submolecules from CS-ESP as further therapeutic molecules. Several proteins, such as enolase, actin, heat shock protein and Ras-related protein, which were found to have diagnostic or pathophysiological value in previous studies, were excluded [18].

We first demonstrated the protective effect of CS-ESP on arthritis. Our data showed a remarkable decrease in IL-17A and GM-CSF production after treatment of CS-ESP in human PBMC, indicating an inhibitory effect of CS-ESP on the systemic circulation. This reduction of inflammatory cytokines was found in both CD4-positive and -negative cells without significant changes in the cell proportions in both RA and AS. This phenomenon might serve as the basis of the beneficial effect of CS-ESP, thus providing protection against immune-mediated diseases. This finding led us to clarify the evidence of therapeutic agents against AS using a murine model. Using a SKG mouse model, we found that the CS-ESP treatment group presented markedly ameliorated disease-specific symptoms. The histological findings supported the inhibitory effect of CS-ESP treatment on arthritis and enthesitis. Our data suggest that CS-ESP has a protective effect against arthritis compared to CSp, as previously demonstrated [7].

After confirming the anti-inflammatory effects using substances secreted by the parasite, we analyzed the components using proteomics and identified two proteins, Cs-GT and Cs-Severin. Of the several proteins among the components of CS-ESP, Cs-Severin and Cs-GT were selected because of their abundance. This was also supported by a previous study on the genome and transcriptome of *C. sinensis* [19, 20]. Finally, we could confirm anti-inflammatory effects of the two proteins. Cs-GT or Cs-Severin single treatment or combination treatment also remarkably decreased IL-17A and GM-CSF production in human PBMC. Concerning the anti-inflammatory benefits in autoimmune conditions like RA and AS, our findings suggest that the activity of *C. sinensis* may actually have therapeutic potential. It seems that *C. sinensis* utilizes specific proteins and metabolic processes to regulate the host's inflammatory response, as shown in Supplementary Figure S2, which could be advantageous in conditions like RA and AS, where excessive immune activity results in tissue damage. The upregulation of detoxifying enzymes, such as Cs-GT, may play a role in reducing oxidative stress, a critical factor driving inflammation in these diseases [21–23]. By neutralizing reactive oxygen species (ROS), *C. sinensis* helps reduce joint inflammation and tissue damage, thus easing symptoms associated with RA and AS. In addition, the increased expression of structural proteins like paramyosin could aid in regulating immune responses by impacting immune cell activation and inflammatory signaling [24–26]. In addition to *C. sinensis* paramyosin, *Trichinella spiralis* paramyosin also exhibited a similar immunoregulatory effect in an arthritis model [27]. Based on this concept, we aimed to explore the other candidates with immunoregulatory effects that could control the excessive immune activity observed in autoimmune conditions, potentially reducing pain, joint stiffness and tissue damage. The function of Cs-Severin in regulating actin dynamics could further enhance the anti-inflammatory effects of *C. sinensis* [20, 28, 29]. By severing and capping actin filaments, Cs-Severin may disrupt the cytoskeletal arrangements of immune cells, thereby limiting their activation and movement toward inflamed sites. This disruption could help reduce immune cell-mediated damage and inflammation, offering an additional mechanism for alleviating symptoms in RA and AS. Therefore, the strategies of *C. sinensis* for mitigating inflammation might be viewed as adaptive mechanisms with potential therapeutic benefits, providing a natural approach to managing chronic inflammatory diseases and enhancing patient outcomes in RA and AS. Recombinant proteins such as Cs-GT and Cs-Severin can be efficiently used in the manufacturing process, aiding industrial production [30]. Data are lacking on specific proteins among the components of CS-ESP. Chen et al. identified Cs-Severin and demonstrated its ability to bind with calcium ions and actin filaments, playing a further role by preventing apoptotic mitochondrial changes (loss of mitochondrial membrane potential) [20]. CS-ESP proteins can play crucial roles against various toxicants, especially in helminth parasites that lack the cytochrome P-450 (CYP450) phase II detoxification enzyme, and might be involved in protecting the reproductive system during maturation of worms [31].

## Conclusion

In this study, we demonstrated that Clonorchis sinensis-Excretory/Secretory Proteins (CS-ESP), along with its components Cs-GT and Cs-Severin, possess significant anti-inflammatory properties that ameliorate clinical symptoms and cytokine abnormalities associated with rheumatoid arthritis (RA) and ankylosing spondylitis (AS). CS-ESP treatment notably reduced the production of pro-inflammatory cytokines such as IL-17A and GM-CSF in peripheral blood mononuclear cells (PBMCs) from RA and AS patients, without affecting cell viability. Furthermore, in vivo experiments using SKG mouse models confirmed that CS-ESP alleviates arthritis and enthesitis. Proteomic analyses identified Cs-GT and Cs-Severin as key proteins with potent anti-inflammatory effects, as evidenced by their ability to suppress inflammatory cytokine production in vitro. Although the lack of in vivo validation for Cs-GT and Cs-Severin is a limitation, our findings highlight their therapeutic potential as promising candidates for the treatment of chronic inflammatory diseases such as RA and AS.

## Data Availability

No datasets were generated or analyzed during the current study.
